# Interpretable machine learning models to predict cadmium in wheat for safe production and soil management

**DOI:** 10.1016/j.fmre.2025.05.001

**Published:** 2025-05-09

**Authors:** Qi-Xin Lü, Zhi-Xian Tang, Zhong Tang, Ge Dong, Zhong-Rui Xu, Fang-Jie Zhao, Peng Wang

**Affiliations:** Center for Agricultural and Environmental Health, Jiangsu Collaborative Innovation Center for Solid Organic Waste Resource Utilization, College of Resources and Environmental Sciences, Nanjing Agricultural University, Nanjing 210095, China

**Keywords:** Cadmium, Wheat, Machine learning, Prediction model, Soil thresholds

## Abstract

Accurate prediction of cadmium (Cd) concentrations in wheat grain is essential for ensuring food safety and sustainable agriculture. Here, we developed a predictive model using nine machine learning (ML) algorithms based on a dataset of 1,339 soil-wheat grain pairs, with a focus on soil properties. The results showed that the eXtreme Gradient Boosting (XGBoost) model outperformed others, achieving superior predictive accuracy (*R*^2^ = 0.90) compared to multiple linear regression (*R*^2^ = 0.69). Through Shapley Additive Explanations (SHAP) analysis, soil total Cd (mean |SHAP| value, 0.20) and pH (0.08) were identified as key determinants, while soil Mn (0.06) and Zn (0.03) concentrations as minor determinants for wheat grain Cd. Soil Cd had a positive effect on grain Cd concentration, whereas soil pH, Mn and Zn showed negative effects. Extending the XGBoost model with 373 nation-scale paired data confirmed its robustness (*R*^2^ = 0.86), and identified high-risk areas for Cd accumulation in southwest China and northwestern Henan province. An online application (https://wheat.cdpredict.cn) was developed for rapid Cd predictions in wheat. To ensure compliance with the wheat grain Cd limit of 0.1 mg/kg, soil Cd safety thresholds were established for different soil pH ranges. We further recommend that approximately 3.4% and 10.5% of cultivated soils should maintain Cd levels within 0.30 and 0.34 mg/kg, respectively. This interpretable ML model provides an actionable tool for managing soil contaminated with Cd to ensure the safe production of wheat.

## Graphical abstract

**Figure:**
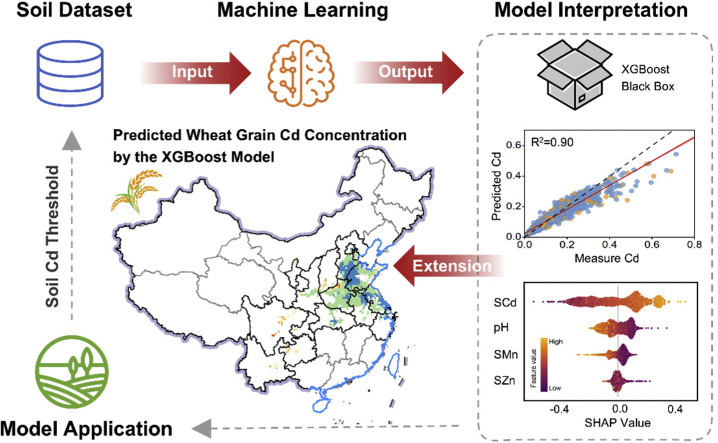

## 1 Introduction

Cadmium (Cd) is a persistent toxic pollutant that poses significant challenges to sustainable agricultural soil management [[1], [2], [3]]. As a major cereal crop, wheat readily accumulates Cd in its grain, posing substantial risks to food safety and public health [4,5]. Globally, wheat is an important contributor to dietary Cd intake [6,7]. Long-term Cd exposure can lead to chronic health issues, particularly in populations reliant on grain-based diets [8,9]. Alarmingly, even in regions considered to be uncontaminated, studies have reported excessive Cd levels in wheat grains, highlighting the pervasive nature of this issue [10,11]. Factors such as soil metal contents, pH, organic matter, water and fertilizer management, and genetic differences among wheat varieties influence the transfer of Cd from soil to crops [[12], [13], [14]]. These complexities underscore the need for reliable predictive models to estimate grain Cd concentrations and identify actionable control strategies to mitigate risks under diverse field conditions.

Previous studies have employed traditional multiple linear regression (MLR) methods to predict Cd accumulation in different parts of wheat [15,16]. These studies often used a single soil Cd measure (total or available) along with soil properties like pH and organic matter for empirical modeling [17,18]. However, such methods rarely consider the nonlinear relationship between grain Cd and various soil factors. Although the multi-surface model (MSM), based on ion adsorption, has been developed to predict Cd availability in wheat field studies [19,20], its predictive accuracy was comparable to that of the MLR model, with limited ability to quantify the contribution of each factor. Recently, machine learning (ML) algorithms, such as random forest (RF), support vector machine (SVM), and artificial neural network (ANN), have gained attention for predicting the content and toxicity of environmental pollutants due to their nonlinear fitting capability and noise immunity [[21], [22], [23]]. Notably, satisfactory Cd prediction models for soil-rice systems have been established using various ML algorithms at different survey scales, with *R*^2^ values ranging from 0.54 to 0.81 [24,25]. Certain ensemble learning algorithms, such as gradient boosting machines (GBM) and eXtreme gradient boosting (XGBoost), have also shown superior generalization in soil Cd prediction [26,27], while cross-validation techniques within the ML training process enhance model robustness [28,29]. Nonetheless, most ML models developed to date are limited to small, localized datasets due to sampling costs and practical operability constraints, resulting in uncertain applicability across broader regions [23,30]. To our knowledge, ML system modeling for predicting wheat grain Cd levels at a nation-wide scale has not been reported.

Establishing an accurate soil Cd safety threshold is crucial for ensuring the safe production of crops [31]. Traditional methods, such as soil-crop transfer linear models and species sensitivity distributions (SSD), have been used to derive these thresholds [10,32]. However, these approaches often rely on data from specific experimental conditions, limiting their applicability across diverse soil types and regions [33,34]. In this study, we leveraged ML algorithms to develop high-precision models for predicting wheat grain Cd concentrations, enabling a more comprehensive understanding of the environmental factors influencing Cd accumulation. By interpreting the internal mechanisms of these ML models, we derived soil Cd thresholds that are both more universal and adaptable to varying conditions. Furthermore, we validated these ML models to ensure their reliability, offering robust support for advancing clean and safe agricultural practices on a large scale.

## 2 Materials and methods

### 2.1 Study areas and sample collection

**Fig. 1:**
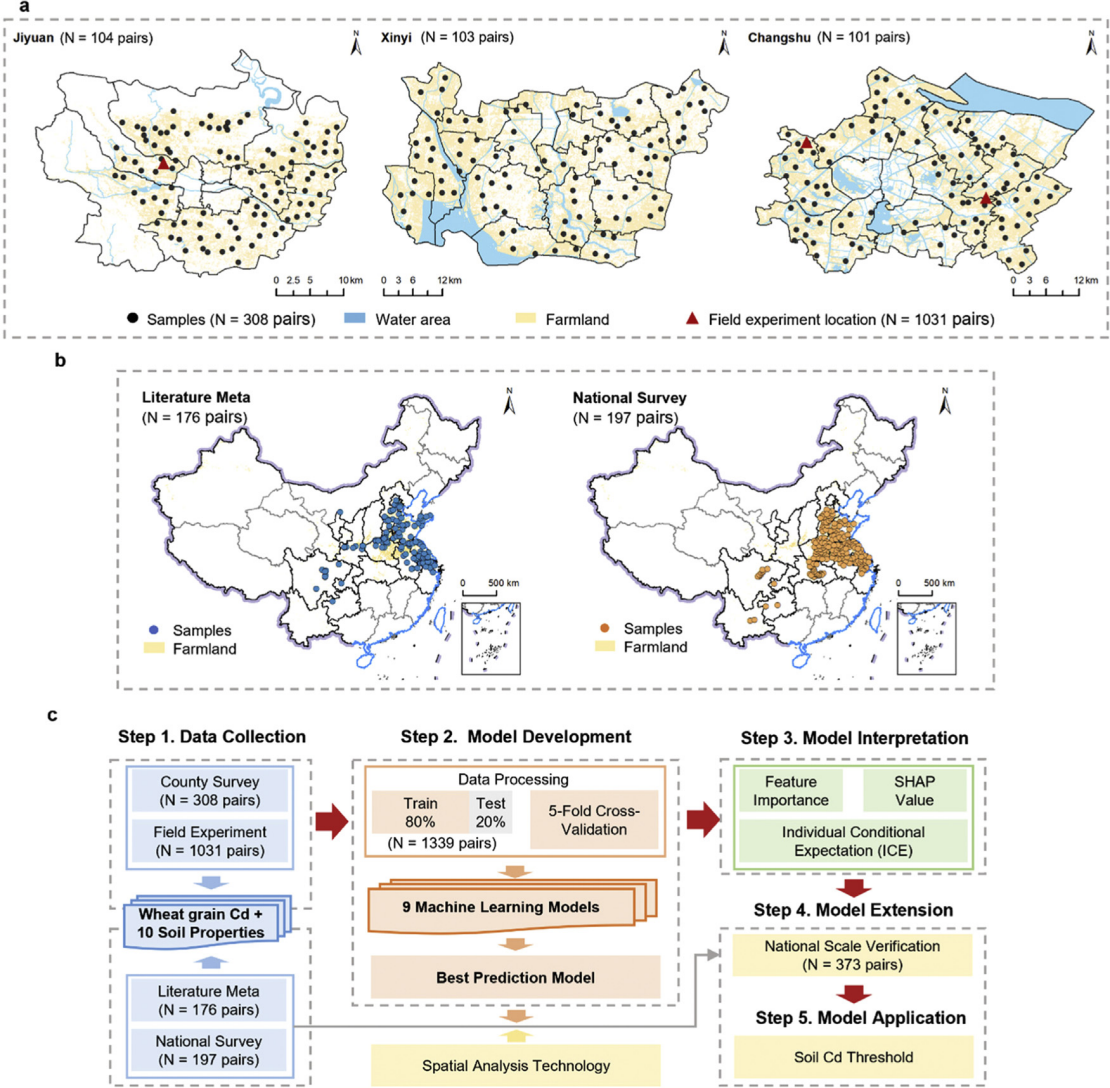
**Overview of the study.** (a) County sampling and field experiments: Locations of Jiyuan, Xinyi, and Changshu counties where soil and wheat grain samples were collected, and field experiments were conducted. (b) Literature meta-analysis and national sampling: Study sites across 17 major wheat-producing provinces in China, incorporating data from literature meta-analyses and national sampling efforts. (c) Analysis workflow: Diagram of the research methodology, illustrating the integration of regional surveys, field experiments, literature meta-analyses, and national sampling into the study's framework. The map of China is edited on the Chinese standard map GS(2019)1697.

This study integrated data from four sources: regional surveys, field experiments, literature meta-analyses, and national-scale sampling (Fig. 1). Three county-scale study areas with diverse climatic and agricultural characteristics were selected: Jiyuan, Xinyi, and Changshu in China (Fig. 1a). Jiyuan, located in northwest Henan province, represents dry farming in northern China, with a wheat cultivation area of 248 km^2^. Xinyi and Changshu, situated in northern and southern Jiangsu province, respectively, have practiced wheat-rice rotation for many years, with wheat cultivation areas of 535 and 180 km^2^. The dominant soil types in these regions are Cambosols and Anthrosols [35]. Prior to the wheat harvest in 2023, 308 pairs of topsoil (0–20 cm) and wheat grain samples were collected: 104 pairs from Jiyuan, 103 pairs from Xinyi, and 101 from Changshu. Sampling locations were recorded using a handheld GPS receiver (Fig. 1a). The sampling points were approximately 3 km apart, and each sample consisted of five paired sub-samples within 0.1 km^2^ of farmland area. Samples were collected in self-sealing bags, with at least 1000 *g* for soil and 200 *g* for wheat grain per sample.

Field experiments were conducted in Jiyuan and Changshu (Fig. 1a), where soils were contaminated with Cd. A randomized block design with four replicates per treatment was employed. Treatments included the applications of various amendments, such as lime, Mn/Zn fertilizers, and organic materials (Table S1). Wheat (cv. Yangmai 23) was grown in 2021 and 2023. At wheat maturity, each 25 m^2^ plot was sampled using a five-point method, yielding 1031 pairs of soil and wheat grain samples from all plots.

Literature meta-analyses and national sampling targeted 17 major wheat-producing provinces across China (Fig. 1b). The meta-analysis included 176 data pairs from peer-reviewed studies published between 2000 and 2023, sourced from the Web of Science and China National Knowledge Infrastructure using keywords “cadmium” and “wheat” or “soil”. The literature screening process is detailed in Fig. S1. An additional 197 data pairs were collected in this study through national sampling in 2024, from the North China Huang-Huai region (*n* = 84), the Middle and Lower Reaches of the Yangtze River region (*n* = 98), and the Southwest region (*n* = 15) (Table S2). Sampling sites were selected via stratified randomization to represent diverse environmental types, but extreme Cd pollution conditions (like farmland near mining areas) were intentionally avoided to reduce assessment uncertainty. Other requirements follow the regional sampling methodology used in 2023.

### 2.2 Chemical analysis

Soil samples were air-dried at room temperature, ground, and sieved through a 10-mesh sieve for physico-chemical property analysis, or a 100-mesh sieve for total metal concentration analysis. Wheat grain samples were dehulled and oven-dried at 60 °C for chemical analysis.

Total concentrations of soil metals (Cd, Mn, Cu, and Zn) were determined after digestion with aqua regia (80/20 HCl/HNO_3_, v/v) using inductively coupled plasma mass spectrometry (ICP-MS, Perkin Elmer NexION 300, USA) [24]. Soil pH (1/2.5 soil/water, w/v) was measured using a glass pH electrode, and soil organic carbon (SOC) was determined using a TOC analyzer (Analytik Jena, multi N/C, Germany) [36]. Wheat grain samples were digested with high-purity concentrated HNO_3_ on a graphite furnace heating block, and Cd concentrations were measured by ICP-MS [24]. Quality control, including procedural blanks, duplicates, and standard reference materials (GBW07428 for soil and GBW10011 for wheat). Indium (In) served as an internal standard for correcting matrix effects. The recovery rates for standard reference materials ranged from 88.6% to 107.8% for soil and 90.2% to 109.5% for grain, with relative standard deviations below 5%.

### 2.3 Establishment of the Cd prediction model

Model development followed the workflow depicted in Fig. 1c. The measured Cd concentration in wheat grain was used as the dependent variable (Table S3). Initial independent variables included ten soil properties: soil pH, SOC, total concentrations of Cd, Mn, Cu, Zn, cation exchange capacity (CEC), and particle size (clay, silt, sand). Of these, CEC and particle size were extracted by ArcGIS 10.2 from the high-resolution China Soil Information Grids Dataset [37]. Stepwise multiple linear regression was employed in feature engineering to select soil variables with higher contributions for the final model [28]. All data except for pH were log-transformed to standardized dimensions.

Data from the regional surveys and field experiments were combined to form the modeling database (Table S3). Using the *sample* function in *R* 4.2.1, 1339 paired data were randomly sampled and divided into train (80%, *n* = 1071) and test sets (20%, *n* = 268) in an 8:2 ratio. Descriptive statistics for the data partitions are presented in Table S4. A 5-fold cross-validation method was applied during training to prevent over-fitting; each fold retained 20% of the training data for validation, while the remaining 80% were used for model training [27,38]. Hyperparameters of each algorithm (Table S5) were tuned via grid search to ensure accurate prediction, and the performance across folds is reported in Table S6.

Nine machine learning algorithms were compared to establish the best prediction model: MLR, generalized linear model (GLM), RF, SVM, ANN, backpropagation neural network (BPNN), GBM, XGBoost, and light gradient boosting machine (LightGBM). These supervised learning models can handle linear and nonlinear regression tasks [21]. Detailed descriptions of these algorithms are provided in the supplementary information (Text S1). Model performance was evaluated using the coefficient of determination (*R*^2^), root mean square error (RMSE), and bias [24,30]. The optimal prediction model was selected based on the highest *R*^2^ value and the lowest loss functions (RMSE, bias) across the full dataset.

### 2.4 Model interpretation and extension

While tree-based models like RF offer inherent feature importance metrics, many machine learning models function as “black boxes”, making their internal decision processes opaque [39,40]. To elucidate the contributions of individual variables in our optimal prediction model, we employed Shapley Additive Explanations (SHAP) [28]. Rooted in cooperative game theory, SHAP assigns a contribution score to each input feature for every prediction, with higher SHAP values indicating greater influence on the model’s output [29]. Additionally, we utilized Individual Conditional Expectation (ICE) plots to visualize feature dependencies. ICE plots illustrate how changes in a feature affect the prediction for individual observations, thereby highlighting heterogeneous effects across samples [41]. The mean line in an ICE plot represents the overall relationship between the dependent variable and the selected feature [42].

To extend the prediction model to a national scale, we incorporated 373 additional sample pairs from across the main wheat-producing areas (Table S7). We evaluated the model’s performance on this expanded dataset using the *R*^2^ value from regression analysis. To enhance the model’s accessibility and usability, we developed a web-based application using Python 3.9.6 and Flask 3.0.1 (https://flask.palletsprojects.com). This platform allows users to input data, receive predictions, and visualize results interactively.

### 2.5 Derivation of soil safety thresholds

In alignment with the current Soil Environmental Quality Standards of China (SEQS, GB 15618–2018), we focused on two key parameters: soil Cd and pH. Utilizing an optimized ML model, we estimated soil Cd safety thresholds across different pH conditions to comply with the wheat grain Cd safety standard of 0.10 mg/kg in China (GB 2762–2022). We validated these proposed thresholds using the four-quadrant method, which involves comparing the ratio of soil Cd concentrations to its threshold value against the ratio of grain Cd content of 0.1 mg/kg [17]. Subsequently, we employed spatial analysis techniques to classify and map soil Cd thresholds for safe wheat production across China.

### 2.6 Statistical analysis

Descriptive statistics and correlation analysis were conducted using SPSS 25.0 (IBM, Armonk, USA). The machine learning workflow was implemented in R Studio. Specifically, the MLR, GLM, RF, SVM, ANN, BPNN, GBM, XGBoost, and LightGBM methods were established using the *lm* function, *caret* package, *randomForest* package, *e1071* package, *nnet* package, *neuralnet* package, *gbm* package, *xgboost* package, and *lightgbm* package, respectively. Cross-validation used the *e1071* package, while feature interpretation was facilitated by the *shapviz* and *DALEXtra* packages. Soil data extraction and map delineation were carried out using ArcGIS 10.2 (Esri, Inc, USA), and graphical representations were created with Origin pro 2021 (Origin Lab, Northampton, MA).

## 3 Results

### 3.1 Soil-wheat grain paired surveys

**Fig. 2:**
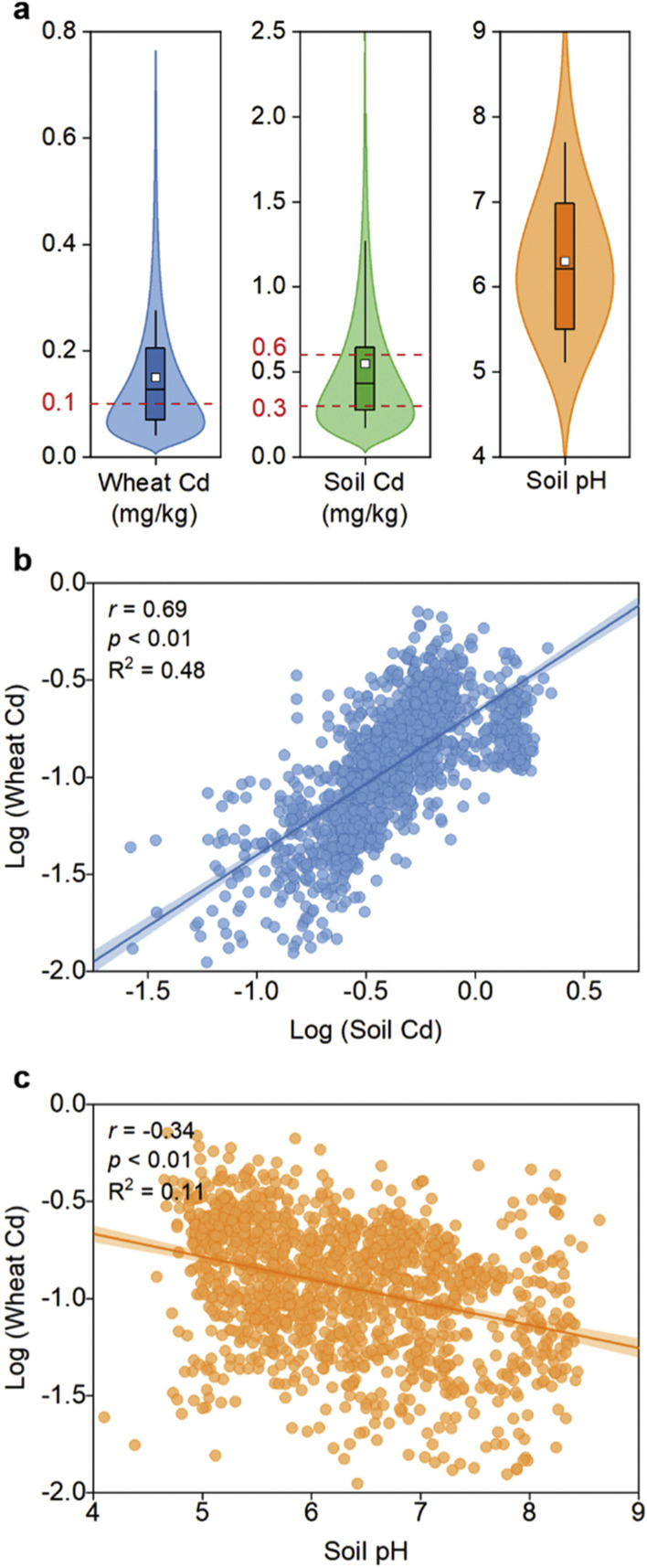
**Statistical analysis of the study.** (a) Statistics plots of wheat grain Cd, soil Cd, and soil pH (*n* = 1339). Squares represent mean values, solid lines indicate median values, and dotted lines denote standard limit values. Correlations between wheat Cd and soil Cd (b) and soil pH (c).

The Cd concentration in wheat grains from regional surveys and field experiments ranged from 0.01 to 0.71 mg/kg (*n* = 1339), with a mean of 0.15 mg/kg and a median of 0.13 mg/kg (Fig. 2a; Table S3). Notably, 818 samples (61.1%) exceeded the Chinese wheat Cd safety limit of 0.10 mg/kg, as stipulated by GB 2762–2022. The coefficient of variation (CV) for grain Cd was 68.9%, indicating moderate variability. Spatially, higher grain Cd concentrations were observed in western Jiyuan, northwestern Xinyi, and both northwestern and southeastern Changshu (Fig. S2).

Soil total Cd concentrations varied widely, ranging from 0.03 to 2.23 mg/kg, with a mean of 0.55 mg/kg and a median of 0.53 mg/kg (Table S3). Approximately 67.4% of the soil samples had Cd concentrations exceeding the soil risk screening values outlined in the SEQS (Fig. 2a). Soil Cd also exhibited a medium variation (CV = 72.9%). Elevated soil Cd concentrations were primarily located in the west of Jiyuan, the northeast of Xinyi, and the northwest and southeast of Changshu (Fig. S2). Other soil elements showed the following variation and mean concentrations: Mn ranging from 138 to 1452 mg/kg with a mean of 391 mg/kg, Cu ranging from 10.2 to 58.0 mg/kg with a mean of 25.1 mg/kg, and Zn ranging from 29.2 to 473 mg/kg with a mean of 88.6 mg/kg (Table S3).

Soil pH values ranged from 4.10 to 8.64, with a mean of 6.30 and a median of 6.21 (Fig. 2a). Lower soil pH levels corresponded spatially with higher soil Cd concentrations in the regional surveys (Fig. S2). Additional soil properties included SOC ranging from 2.13 to 56.3 *g*/kg, CEC from 14.2 to 23.3 cmol/kg, and particle size ratio distributions with sand, clay, and silt contents ranging from 8.60% to 32.8%, 15.3% to 30.7%, and 44.4% to 67.9% respectively (Table S3).

A moderate positive correlation was found between wheat grain Cd and soil Cd concentrations, with a correlation coefficient of 0.45, which increased to 0.69 after logarithmic transformation, yielding an *R*^2^ value of 0.48 (Fig. 2b). Soil pH exhibited a negative correlation with wheat grain Cd, with a correlation coefficient of −0.34 and an *R*^2^ of 0.11 (Fig. 2c). Soil Mn and Zn contents also showed absolute correlation coefficients of ≥ 0.20 with wheat grain Cd concentrations (Fig. S3).

### 3.2 Prediction model of grain Cd concentration

Stepwise multiple linear regression was conducted to assess the impact of ten soil characteristics on grain Cd levels. Soil total Cd alone explained 47.8% of the variance in grain Cd concentration (Eq. 1 in Table S8). Incorporating soil pH increased the explanatory power to 64.8% (Eq. 2). The optimal linear regression model combining soil total Cd, pH, total Mn, and total Zn produced an *R*^2^ of 0.70 (Eq. 4). This aligns with the correlation analysis results (Fig. S3), suggesting that the other six variables contributed minimally. Consequently, soil total Cd, pH, total Mn, and total Zn were selected as final input features for machine learning modeling.

**Fig. 3:**
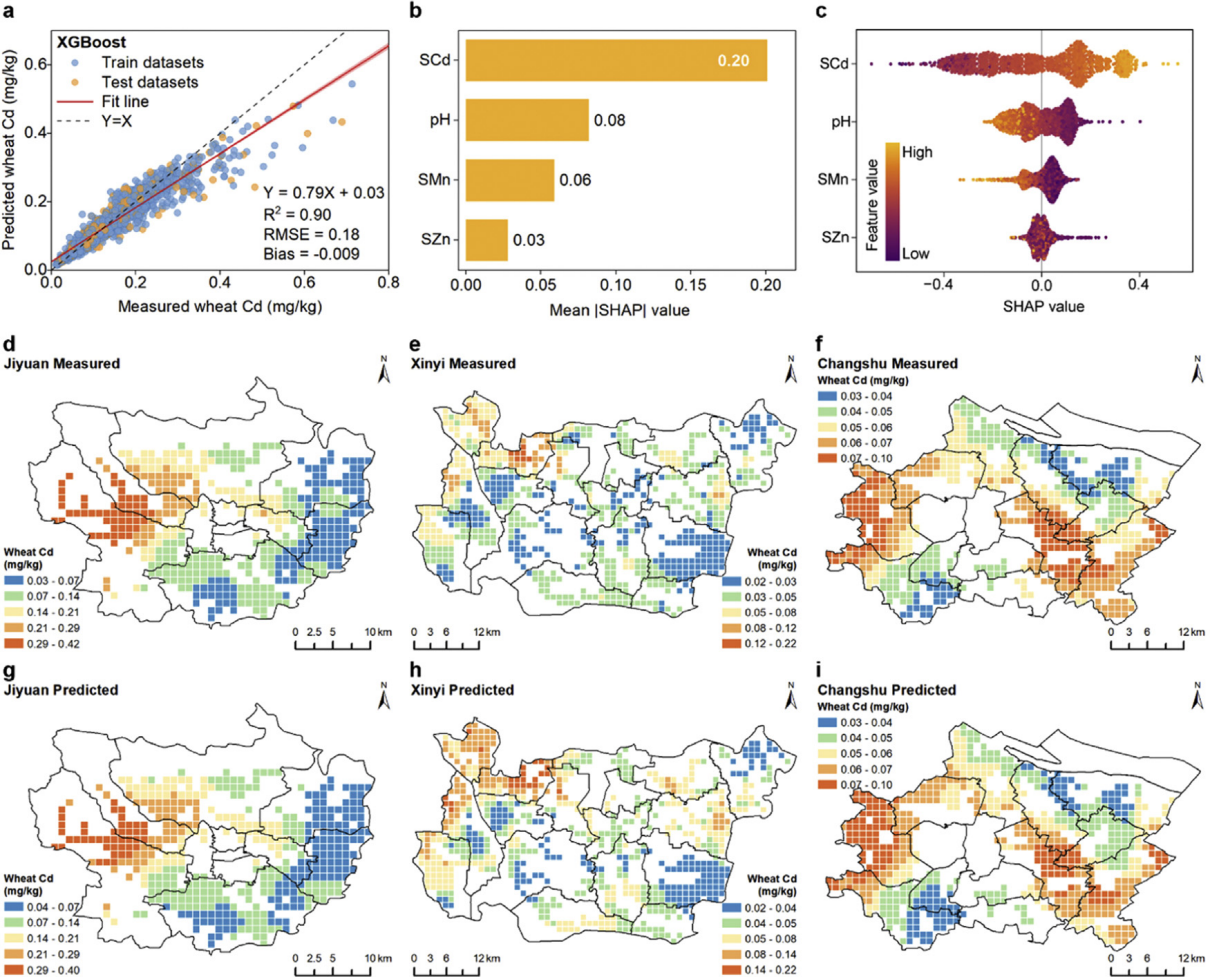
**Prediction of wheat grain Cd concentrations based on the XGBoost model.** (a) Scatter plot of measured versus predicted wheat grain Cd concentrations (*n* = 1339, train: test = 8:2). (b) Bar plot of mean absolute SHAP values, indicating feature impact. (c) SHAP summary plot, where each point represents an individual sample, colored by feature magnitude (high: orange; low: purple). Spatial distribution maps of measured (d-f) and predicted (g-i) Cd concentrations in wheat grains across the study areas. The map of China is edited on the Chinese standard map GS(2019)1697.

A dataset of 1071 data pairs (80% of the total, Table S4) was used for model training. Performance comparisons among nine machine learning algorithms are presented in Fig. S4 and Table S9. The models ranked by *R*^2^ on the training sets were as follows: XGBoost (0.95) > RF (0.94) > LightGBM (0.85) > GBM (0.79) > BPNN (0.75) > ANN (0.73) > SVM (0.70) > GLM (0.69) > MLR (0.69). On the test sets, the XGBoost model demonstrated superior performance, with an *R*^2^ of 0.88, RMSE of 0.169, and bias of 0.012. The RF algorithm followed closely (*R*^2^ = 0.85, RMSE = 0.171, bias = 0.013). Linear models exhibited lower accuracy, with *R*^2^ values of 0.55 and 0.56 for MLR and GLM, respectively. Overall, the XGBoost model provided the most accurate predictions for wheat grain Cd, achieving an *R*^2^ of 0.90 on the full dataset (Fig. 3a).

The XGBoost model’s feature importances were analyzed using SHAP values, which quantify each feature’s contribution to the model’s predictions. The analysis revealed that soil total Cd (SCd) was the most influential variable on grain Cd levels, with a mean absolute SHAP value of 0.20. This was followed by soil pH (0.08), soil total Mn (SMn, 0.06), and soil total Zn (SZn, 0.03) (Fig. 3b). This ranking indicates that SCd has the strongest impact on grain Cd concentration, followed by pH, SMn, and SZn.

The SHAP summary plot illustrates the effect of each feature across all data points (Fig. 3c). Data points with higher SCd values predominantly have positive SHAP values, indicating that increased SCd contributes to higher grain Cd concentrations. In contrast, higher soil pH and SMn values are associated with negative SHAP values, suggesting that these features contribute to lower grain Cd levels. SZn shows a minor negative impact. The ICE plots further elucidate the relationships between each feature and grain Cd concentration. These plots demonstrate that grain Cd content generally increases with higher SCd levels and decreases with higher soil pH and SMn levels (Fig. S5). This aligns with the SHAP analysis, reinforcing the identified feature contributions.

For external validation, the XGBoost model predicted mean grain Cd concentrations of 0.12 mg/kg for Jiyuan, 0.04 mg/kg for Xinyi, and 0.06 mg/kg for Changshu in regional surveys (*n* = 308). These predictions closely match the observed values (Fig. S6). Additionally, the spatial distributions of predicted grain Cd (Fig. 3d–F) align well with the measured distributions (Fig. 3 g–i) in the three regional surveys, confirming the model’s predictive accuracy.

### 3.3 Extrapolation of predictive models

**Fig. 4:**
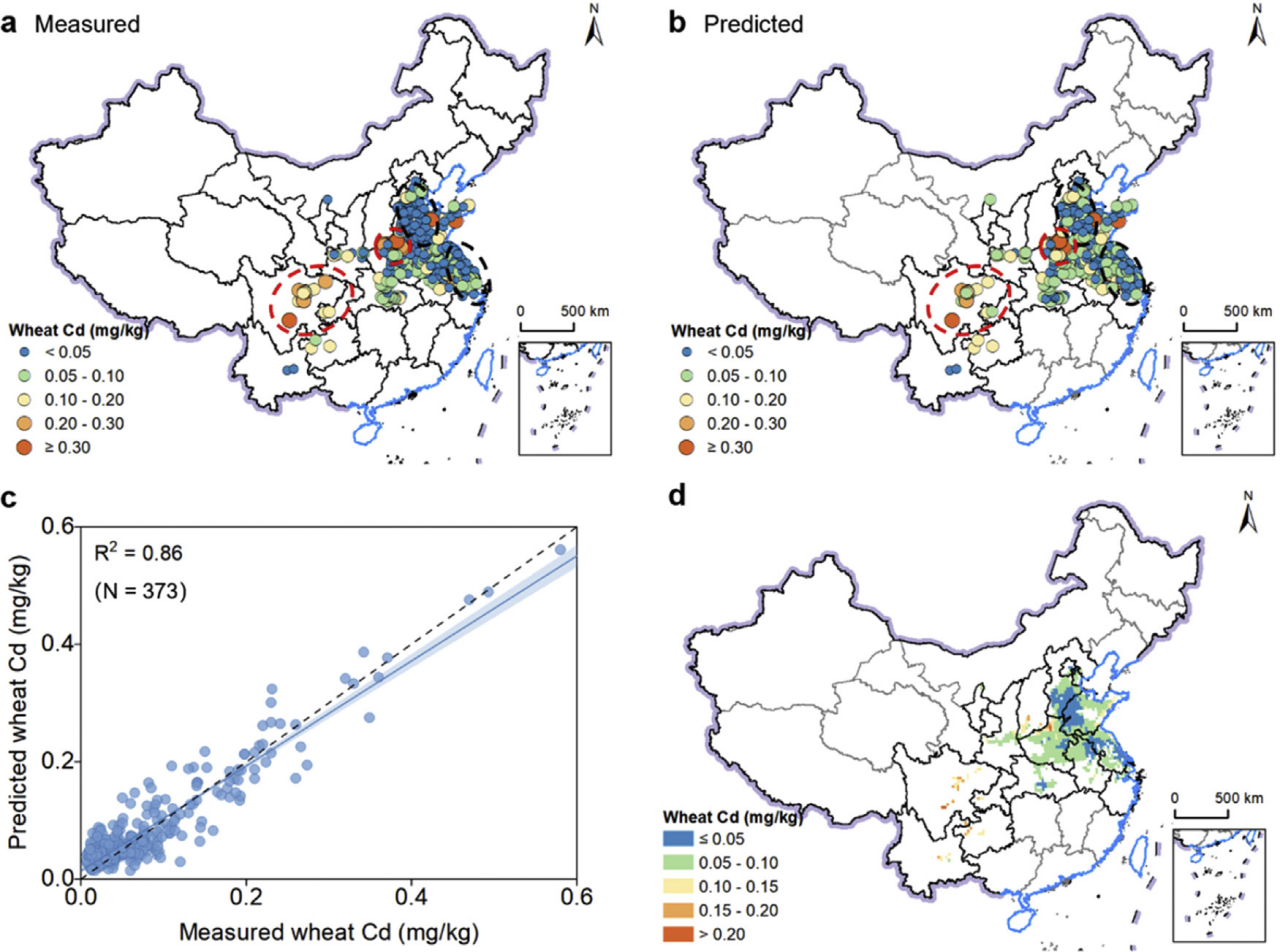
**Extrapolation of the XGBoost model.** Measured (a) and predicted (b) wheat grain Cd concentrations at sampling points. (c) Scatter plots of measured versus predicted Cd concentrations. (d) Predicted spatial distribution maps of wheat grain Cd concentrations in wheat-growing regions in China. The map of China is edited on the Chinese standard map GS(2019)1697.

The XGBoost model was applied to predict Cd concentrations in wheat grains using 373 pairs of samples collected nationwide. The spatial distribution patterns of Cd concentrations in the predicted maps closely matched those of the measured data (Fig. 4a–b). The model predicted grain Cd concentrations ranged from 0.01 to 0.56 mg/kg, with a mean of 0.08 mg/kg and a median of 0.06 mg/kg. Approximately 49.9% of predicted cases had a grain Cd concentration ≤ 0.05 mg/kg, compared to 56.3% in the measured samples. Both predicted and measured datasets showed similar proportions (19.0%) of samples exceeding the 0.1 mg/kg Cd limit. The regression analysis between predicted and measured grain Cd values yielded an *R*^2^ of 0.86 (Fig. 4c), which was only slightly lower than the *R*^2^ value (0.90) of the original modeling (*n* = 1339). Using spatial interpolation techniques, the national predicted Cd concentrations in wheat grains ranged from 0.02 to 0.32 mg/kg, with both mean and median values of 0.06 mg/kg. Regions with potential Cd exceedance (> 0.1 mg/kg) were primarily located in the wheat-growing areas of southwest China and northwestern Henan province, accounting for approximately 12.2% of the total wheat cultivation area (Fig. 4d).

An online application based on the XGBoost model is available at https://wheat.cdpredict.cn. This user-friendly tool provides rapid predictions of wheat grain Cd concentrations, including mean, median, distribution histogram, and exceedance risk probability. Users can input key soil properties such as soil Cd, pH, Mn, and Zn. If soil Mn and Zn data are unavailable, the application operates using default values representing averages.

### 3.4 Soil safety thresholds for Cd

**Fig. 5:**
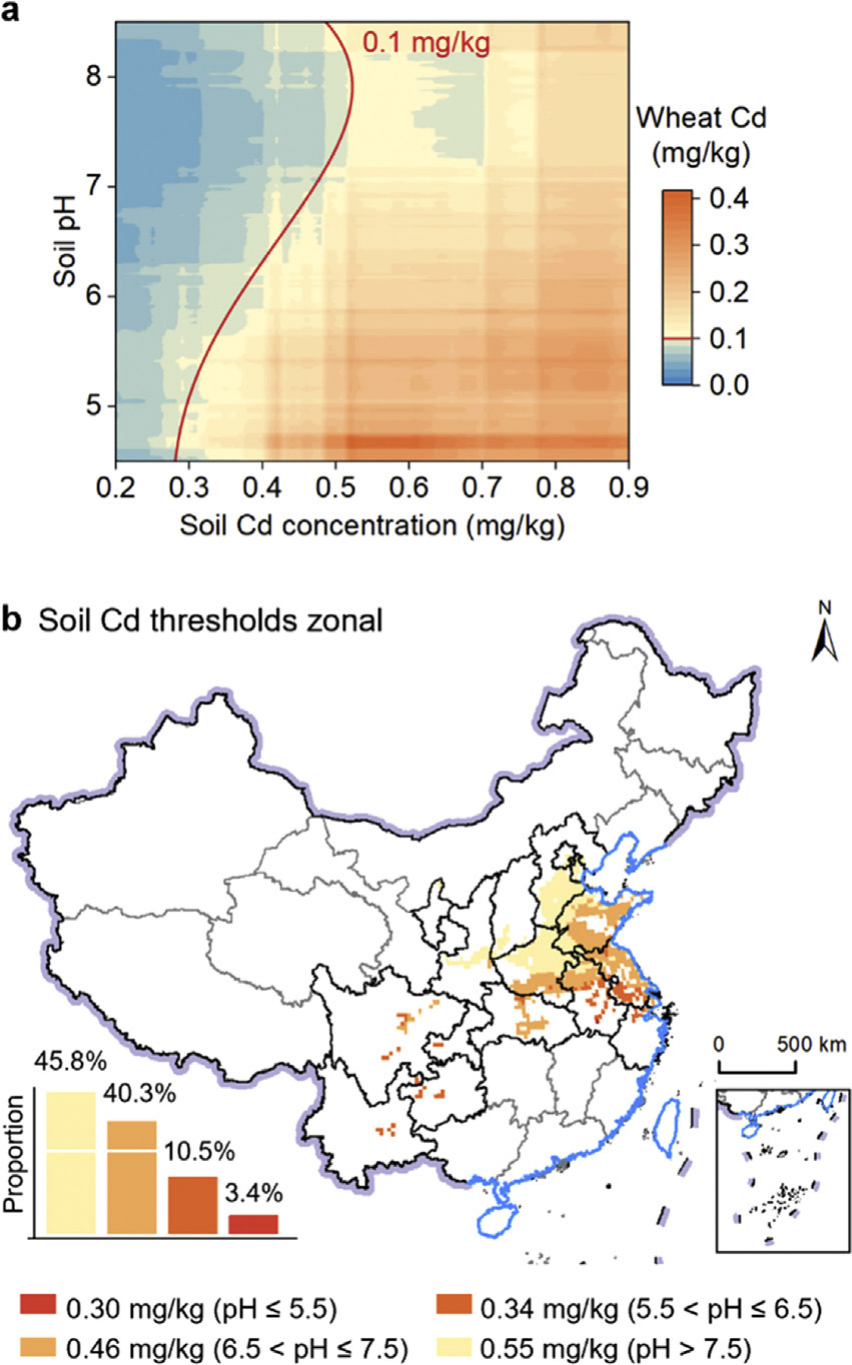
**Soil Cd thresholds for safe wheat production.** (a) Heatmap illustrating predicted wheat grain Cd concentrations as a function of soil Cd concentrations and soil pH values; the red contour line marks the safety threshold of 0.1 mg/kg for wheat grain Cd. (b) Map highlighting spatial distribution of soil Cd thresholds in China, with a bar plot depicting the proportion of total wheat cultivated land area corresponding to each Cd threshold category. The map of China is edited on the Chinese standard map GS(2019)1697.

Using the established XGBoost model, wheat grain Cd were predicted under various combinations of soil Cd levels and pH values (Fig. 5a). Based on these predictions, the safety thresholds of soil Cd to comply with the wheat grain Cd limit were established as follows: 0.30 (95% confidence interval: 0.28–0.32) mg/kg for pH ≤ 5.5, 0.34 (0.32–0.37) mg/kg for 5.5 < pH ≤ 6.5, 0.47 (0.38–0.53) mg/kg for 6.5 < pH ≤ 7.5, and 0.55 (0.51–0.59) mg/kg for pH > 7.5 (Table S10).

Evaluation with the four-quadrant method showed that the model-derived soil thresholds reduced the false negative rate to 9.39% (quadrants Ⅱ: 6.39%, quadrants Ⅳ: 3.00%), improving upon the 11.84% false negative rate associated with the risk screening values in the SEQS (Fig. S7). Applying these soil thresholds to the three study areas revealed that only 2.60% of samples exceeding the grain Cd standard were not identified (Fig. S8). At a national level, it was estimated that 45.8% of cultivated soils would need to limit soil Cd concentrations to below 0.55 mg/kg to meet wheat safety standards. Furthermore, 3.4% and 10.5% of cultivated soils would need to limit Cd concentrations below 0.30 and 0.34 mg/kg, respectively, depending on local soil pH conditions (Fig. 5b). These findings highlight the usefulness of the XGBoost-derived thresholds in supporting targeted and practical soil management strategies for safe wheat production.

## 4 Discussion

### 4.1 Advancing predictive modeling of Cd in wheat grains

This study highlights a significant advancement in predictive modeling by integrating traditional and machine learning approaches to forecast Cd levels in wheat grains. Unlike linear models such as MLR, which showed limited accuracy (*R*^2^ = 0.69) due to their inability to capture nonlinear relationships, the XGBoost algorithm achieved superior predictive performance (*R*^2^ = 0.90) by effectively modeling complex soil-crop interactions [43,44]. The interpretability of the XGBoost model, enhanced through SHAP analysis and ICE plots [29,45], identified soil Cd and pH as the most influential factors driving Cd accumulation. Its scalability and robustness, demonstrated by the consistent performance at the national level (*R*^2^ = 0.86), establish the XGBoost model as a generalizable and reliable tool for large-scale agricultural assessments. Previous research at the county scale reported low predictive accuracy (*R*^2^ = 0.4–0.6) even when additional variables were included in linear models [13,20]. In contrast, the XGBoost algorithm's ensemble learning framework reduces bias and variance. Its built-in regularization techniques, cross-validation, and early stopping mechanisms helped prevent overfitting and ensure robustness against noise and outliers [43,46,47]. The XGBoost model's parallelization and distributed computing capabilities enabled fast training even with large datasets [44]. These attributes allow it to dynamically adapt to varying soil properties, advancing the knowledge frontier in environmental modeling by overcoming the limitations of traditional approaches.

Nevertheless, several limitations should be acknowledged to guide future refinements. The current XGBoost model takes the total metal amount as input after feature engineering; a lack of consideration of bioavailability may lead to potential overestimation in predictions [28]. The wheat varieties used for modeling in the study were limited, and incorporating genotypic differences could further improve prediction accuracy [48]. The performance in extreme regions of soil properties (e.g., saline-alkaline soils with pH > 9 in northwestern China, or contaminated soils with Cd > 5 mg/kg) remains to be verified, as such scenarios are underrepresented in our sampling framework. Addressing these limitations through multi-parameter models that integrate bioavailability, genetic traits, and dynamic environmental variables represents a critical next step for precision forecasting.

### 4.2 Insights into the soil factors affecting grain Cd

This study identifies several key soil factors influencing Cd accumulation in wheat grains. Primarily, soil total Cd concentration emerged as the most significant determinant, aligning with previous research that correlates higher soil Cd levels with increased grain Cd content [14,49]. Soil pH also plays a crucial role; lower pH enhances Cd solubility and uptake by wheat plants [18,50], whereas higher pH promotes Cd adsorption onto iron (Fe) and Mn oxides, thereby reducing its bioavailability [51,52]. Additionally, soil Mn and Zn concentrations were found to affect Cd uptake, likely through competitions for the shared membrane transporters in the roots, such as the Nramp family of transporters [53,54]. However, in rice cultivation, our previous research has highlighted soil pH as the primary determinant of Cd levels in rice grain, followed by Fe/Mn oxide-bound Cd and Mn content [24]. This distinction is attributed to the unique flooding and drainage conditions inherent in paddy fields. During drainage, soil pH regulates the partitioning of Cd between soil particles and the solution phase, affecting its availability to rice plants [55,56]. Fe/Mn oxide-bound Cd represents a pool of Cd that becomes releasable upon soil oxidation [57,58]. Furthermore, Zn and Mn contribute to Cd dynamics through voltaic-cell effects and by competitions for the Nramp5 transporter, which is responsible for Cd and Mn uptake in rice [59,60].

The different factors influencing Cd accumulation in wheat and rice necessitate tailored agronomic strategies for each crop. For wheat, effective management should focus on reducing soil Cd content. Implementing deep tillage can help lower Cd concentrations in the topsoil, while removing Cd-laden crop residues can prevent further contamination [61]. Phytoremediation, utilizing plants that accumulate Cd, offers a sustainable approach to extract Cd from the soil [62]. Balanced fertilization with Mn and Zn can also mitigate Cd uptake by wheat, particularly in alkaline soils [63]. Unlike wheat soil, which is under oxidative conditions for a long time, paddy soil has a significant redox effect, and the absorption of Cd by rice is more responsive to pH than that of wheat. So, rice cultivation should prioritize maintaining optimal pH and redox conditions during paddy field drainage. Applying alkaline materials, such as lime, has been shown to significantly reduce Cd content in rice grains and offers economic benefits [64,65]. Various water management strategies, like intermittent irrigation, also regulate the bioavailability of Cd to mitigate Cd accumulation [56,66]. Notably, wheat exhibits limited genetic variation in grain Cd accumulation (3–4-fold) [48,67], which constrains the potential for varietal improvement. Conversely, rice shows greater genetic variability in Cd accumulation (exceeding 20-fold) [16,68], presenting more opportunities for developing low-Cd cultivars.

### 4.3 Applicability of soil Cd thresholds for safe wheat production

This study established soil Cd safety thresholds tailored to specific pH levels using the XGBoost model, addressing a critical gap in large-scale agricultural management. Unlike traditional species sensitivity distribution methods, which often require complex toxicological analyses, the XGBoost-derived thresholds dynamically adjust to varying soil conditions, offering more precise and adaptable guidelines for sustainable farming [10,69]. This adaptability is particularly beneficial in areas with acidic soils, where Cd risks are heightened [70]. Implementing these tailored thresholds enables effective zoning for wheat cultivation, optimizing resource allocation and reducing economic burdens associated with soil remediation. Furthermore, agronomic practices such as applying alkaline amendments to increase soil pH, balancing micronutrient fertilizers, and selecting Cd-resistant wheat varieties can be strategically implemented based on model predictions, fostering cleaner and safer agricultural production.

## 5 Conclusion

This study established a wheat grain Cd concentration prediction model based on machine learning, with the best performance of the XGBoost method, which showed obvious predictive robustness at a large scale. Soil Cd and pH were key factors influencing grain Cd accumulation, followed by soil Mn and Zn. An online application was further developed, which allows users to predict grain Cd concentrations and assess compliance with safety standards in real time. By developing an interpretable ML model and establishing soil Cd safety thresholds tailored to different pH levels, this research offers new insights for farmers, agronomists, and policymakers to promote clean wheat production. This innovative, data-driven approach addresses the challenges of Cd contamination, supporting sustainable agriculture and food safety on a national scale that can be further extended to the global scale.

## Supporting Information

Further details on descriptive statistics and spatial distributions of the regional survey; additional details on field experiments, literature meta-analyses, and national-scale sampling; results of linear regression analysis; theoretical methods of machine learning, modeling datasets and parameters, accuracy for 5-fold cross-validation, assessment of predict model performance, as well as ICE plots; and statistics of wheat Cd contents under the recommended soil Cd threshold.

## CRediT authorship contribution statement

**Qi-Xin Lü:** Data curation, Formal analysis, Investigation, Methodology, Writing – original draft. **Zhi-Xian Tang:** Investigation, Methodology. **Zhong Tang:** Investigation, Methodology. **Ge Dong:** Investigation, Methodology. **Zhong-Rui Xu:** Investigation. **Fang-Jie Zhao:** Resources, Supervision. **Peng Wang:** Conceptualization, Funding acquisition, Project administration, Supervision, Writing – original draft, Writing – review & editing.

## **Declaration of competing interest**

The authors declare that they have no conflicts of interest in this work.

## Appendix Supplementary materials

Image, application 1
