# Supplementary material for: Interpretable machine learning models to predict cadmium in wheat for safe production and soil management
Source: Fundam Res. 2025 May 9;6(3):1492–500. doi: 10.1016/j.fmre.2025.05.001 (PMC13247447; doi:10.1016/j.fmre.2025.05.001)
Supplement: Supplementary file 1 [file mmc1.docx]

**Supporting Information**

**Interpretable machine learning models to predict cadmium in wheat for safe production and soil management**

Total Pages: 16

Text: 1

Figures: 8

Tables: 10

## Supplementary Text:

**Text S1. Machine learning methods**

**S1.1. Multiple linear regression model (MLR)**

MLR is used to model the relationship between multiple independent variables and a single dependent variable. It assumes a linear relationship between the independent variables and the dependent variable, aiming to find the best-fitting line between the observed values and the predicted values [1]. MLR estimates the coefficients for each input to quantify their impact on the output while considering the effect of other variables in the model [2].

**S1.2. Generalized linear model (GLM)**

GLM is a flexible extension of linear regression that allows for non-normally distributed response variables, such as count, or categorical data [3]. In GLM, the relationship between the mean of the response variable and the predictors is modeled through a link function, which connects the linear predictor to the expected value of the response variable. The specific GLM consists of a random component, a system component, and a coupling function [4].

**S1.3. Random forest (RF)**

RF is an ensemble algorithm that builds multiple decision trees by the bootstrap method during training and outputs the average prediction of individual trees for regression tasks [5]. Each tree in an RF is built using a random subset of the training data and a random subset of features, which helps to reduce overfitting and improve generalization performance [6]. By combining the predictions of multiple trees, RF can provide robust and accurate predictions.

**S1.4. Support vector machine (SVM)**

SVM is a supervised algorithm, which works by finding the optimal hyperplane that best separates the data points into different classes in a high-dimensional space [7]. SVM aims to maximize the margin between the classes, and it can handle non-linear relationships by using kernel functions to map the input data into a higher-dimensional space [8]. The common kernel functions are linear, polynomial, sigmoid, and radial basis functions (RBF).

**S1.5. Artificial neural network (ANN)**

ANN is an algorithm that consists of input, hidden, and output layers, each with a set of interconnected nodes (neurons) working in parallel to transform the input data into output values [9]. The number of neurons in the input layer is equal to the number of the predictors, while the neurons in the hidden layer are determined by calculating the training and prediction errors [10]. Here, ANN adjusts the weights by forward propagation to minimize the difference.

**S1.6. Backpropagation neural network (BPNN)**

BPNN is a type of ANN in which the main algorithm adjusts the weights and biases of the network by propagating the error backward from the output layer to the input layer [11]. This process involves computing the gradient of the loss function for the weights and biases and using this information to gain the minimum error sum of squares through gradient descent or similar optimization algorithms [12].

**S1.7. Gradient boosting machine (GBM)**

GBM is an algorithm that builds an ensemble of decision trees, where each tree corrects the errors made by the previous one [13]. It minimizes prediction errors by optimizing a loss function through gradient descent. The final prediction is a weighted sum of the predictions from multiple weak learners [14]. GBM can capture non-linear relationships and interactions between variables effectively, with high predictive accuracy and robustness against overfitting.

**S1.8. eXtreme gradient boosting (XGBoost)**

XGBoost is an optimized and scalable implementation of the GBM, which builds an ensemble of decision trees sequentially to minimize prediction errors [15]. It employs advanced techniques such as parallelization, tree pruning, and regularization for speed and performance [16]. This integration empowers XGBoost to effectively handle large-scale and high-dimensional datasets, while simultaneously mitigating the risk of overfitting [17]. Additionally, XGBoost showcases the capability to automatically manage missing values.

**S1.9. Light gradient boosting machine (LightGBM)**

LightGBM is a high-performance, distributed gradient-boosting framework. It uses a novel technique called Gradient-based One-Side Sampling (GOSS) to filter out data instances during tree learning, reducing training time while maintaining accuracy [18]. LightGBM also implements features such as histogram-based splitting and leaf-wise growth to further enhance training speed and scalability [19].

**References**

1. T.T. Mu, T. Zhou, Z. Li, et al., Prediction models for rice cadmium accumulation in Chinese paddy fields and the implications in deducing soil thresholds based on food safety standards, Environ. Pollut. 258 (2020) 113879.
2. J. Ran, D.J. Wang, C. Wang, et al., Heavy metal contents, distribution, and prediction in a regional soil-wheat system, Sci. Total Environ. 544 (2016) 422-431.
3. J.A. Nelder, R.W. Wedderburn, Generalized linear models, J. R. Stat. Soc. A Stat. 135 (1972) 370-384.
4. H.R. Chen, L. Wang, B.F. Hu, et al., Potential driving forces and probabilistic health risks of heavy metal accumulation in the soils from an e-waste area, southeast China, Chemosphere 289 (2021) 133182.
5. L. Breiman, Random Forests, Machine Learning, 45 (2001) 5-32.
6. A. Cutler, D.R. Cutler, J.R. Stevens, Random Forests, Ensemble machine learning: Methods and applications (2012) 157-175.
7. M.A. Hearst, S.T. Dumais, E. Osuna, et al., Support vector machines, IEEE Intell. Syst. 13 (1998) 18-28.
8. X.L. Jia, B.F. Hu, B.P. Marchant, et al., A methodological framework for identifying potential sources of soil heavy metal pollution based on machine learning: A case study in the Yangtze Delta, China, Environ. Pollut. 250 (2019) 601-609.
9. W.S. McCulloch, W. Pitts, A logical calculus of the ideas immanent in nervous activity, B. Math. Biol. 52 (1990) 99-115.
10. B. Zhao, W.X. Zhu, S.F. Hao, et al., Prediction heavy metals accumulation risk in rice using machine learning and mapping pollution risk, J. Hazard. Mater. 448 (2023) 130879.
11. D.E. Rumelhart, G.E. Hinton, R.J. Williams, Learning representations by back-propagating errors, Nature 323 (1986) 533-536.
12. A. Mouazen, B. Kuang, J. De Baerdemaeker, et al., Comparison among principal component, partial least squares and back propagation neural network analyses for accuracy of measurement of selected soil properties with visible and near infrared spectroscopy, Geoderma 158 (2010) 23-31.
13. J.H. Friedman, Greedy function approximation: a gradient boosting machine, Ann. Stat. (2001) 1189-1232.
14. G. Ridgeway, Generalized Boosted Models: A guide to the gbm package, Update (2007) 2007.
15. T.Q. Chen, C. Guestrin, Xgboost: A scalable tree boosting system, Proceedings of the 22nd acm sigkdd international conference on knowledge discovery and data mining, (2016) 785-794.
16. J.W. Huang, G.P. Fan, C. Liu, et al., Predicting soil available cadmium by machine learning based on soil properties, J. Hazard. Mater. 460 (2023) 132327.
17. O. Sagi, L. Rokach, Approximating XGBoost with an interpretable decision tree, Inform. Sci. 572 (2021) 522-542.
18. G.L. Ke, Q. Meng, T. Finley, et al., Lightgbm: A highly efficient gradient boosting decision tree, Adv. Neural Inf. Process. Syst. 30 (2017).
19. I.D. Mienye, Y. Sun, A survey of ensemble learning: Concepts, algorithms, applications, and prospects, IEEE Access 10 (2022) 99129-99149.

## Supplementary Figures:


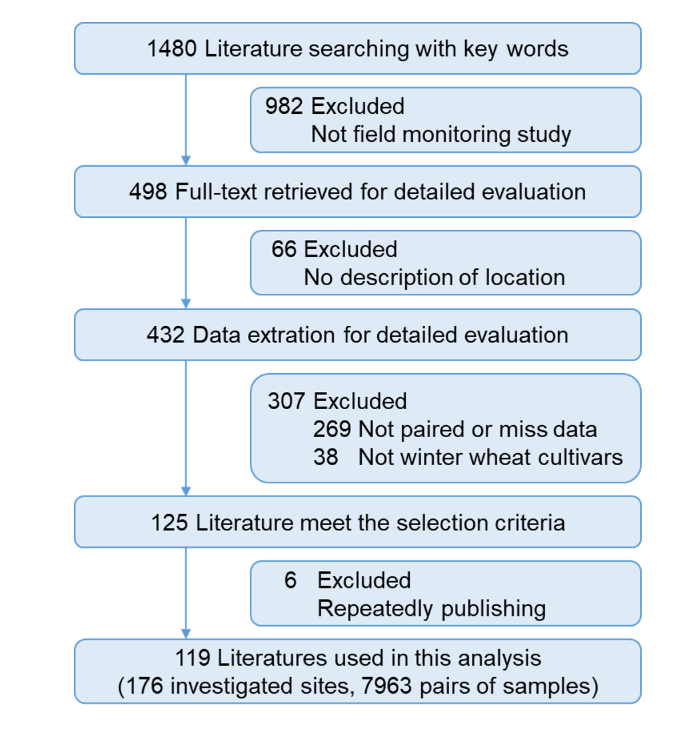


**Fig. S1.** Selection flow chart of published literature from 2000 to 2023.


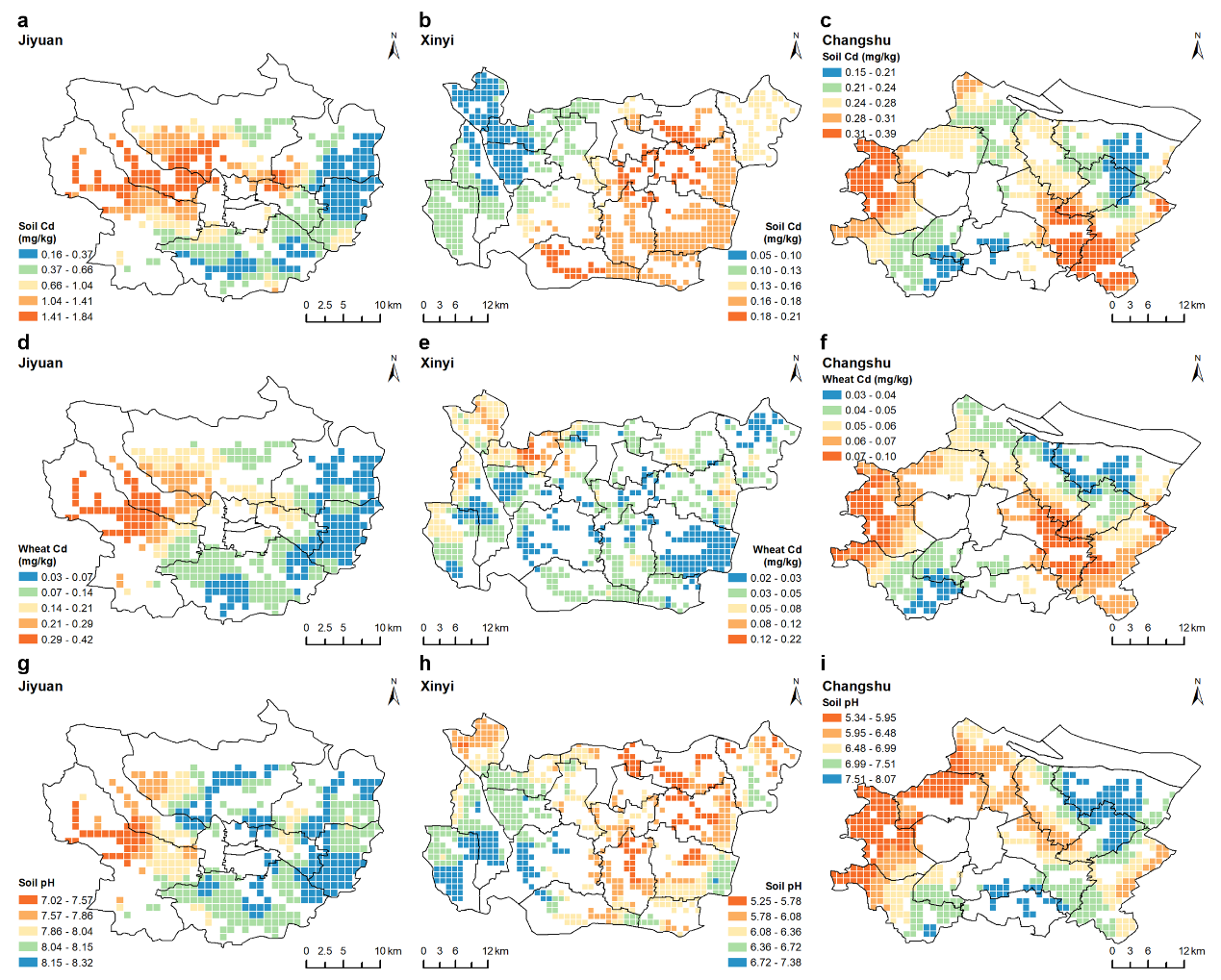


**Fig. S2.** Spatial distribution maps of soil Cd concentration (a-c), wheat grain Cd concentration (d-f), and soil pH (g-i) in the regional surveys.


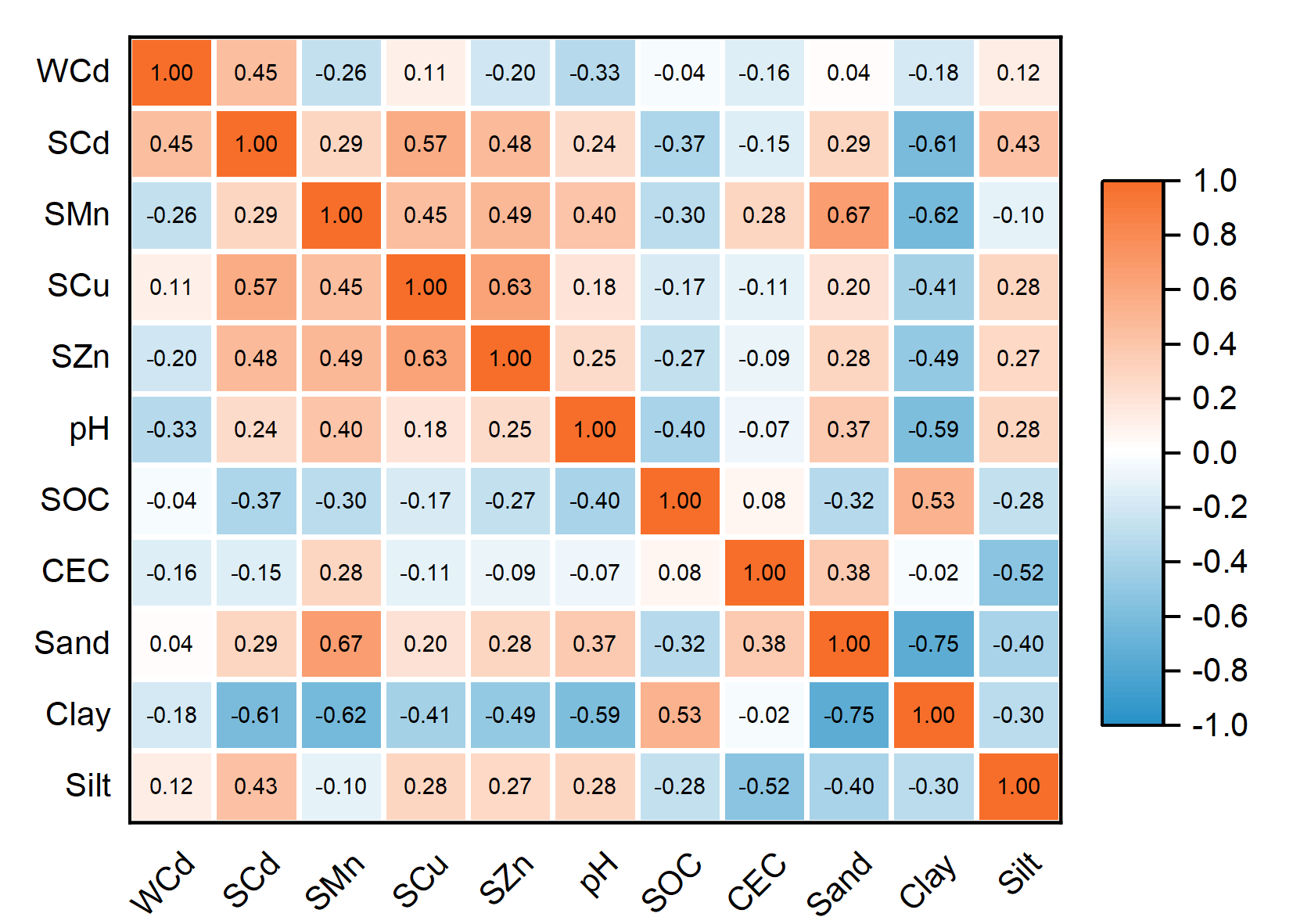


**Fig. S3.** Correlation analysis of wheat grain Cd, soil metals, and soil physico-chemical properties (n = 1,339).


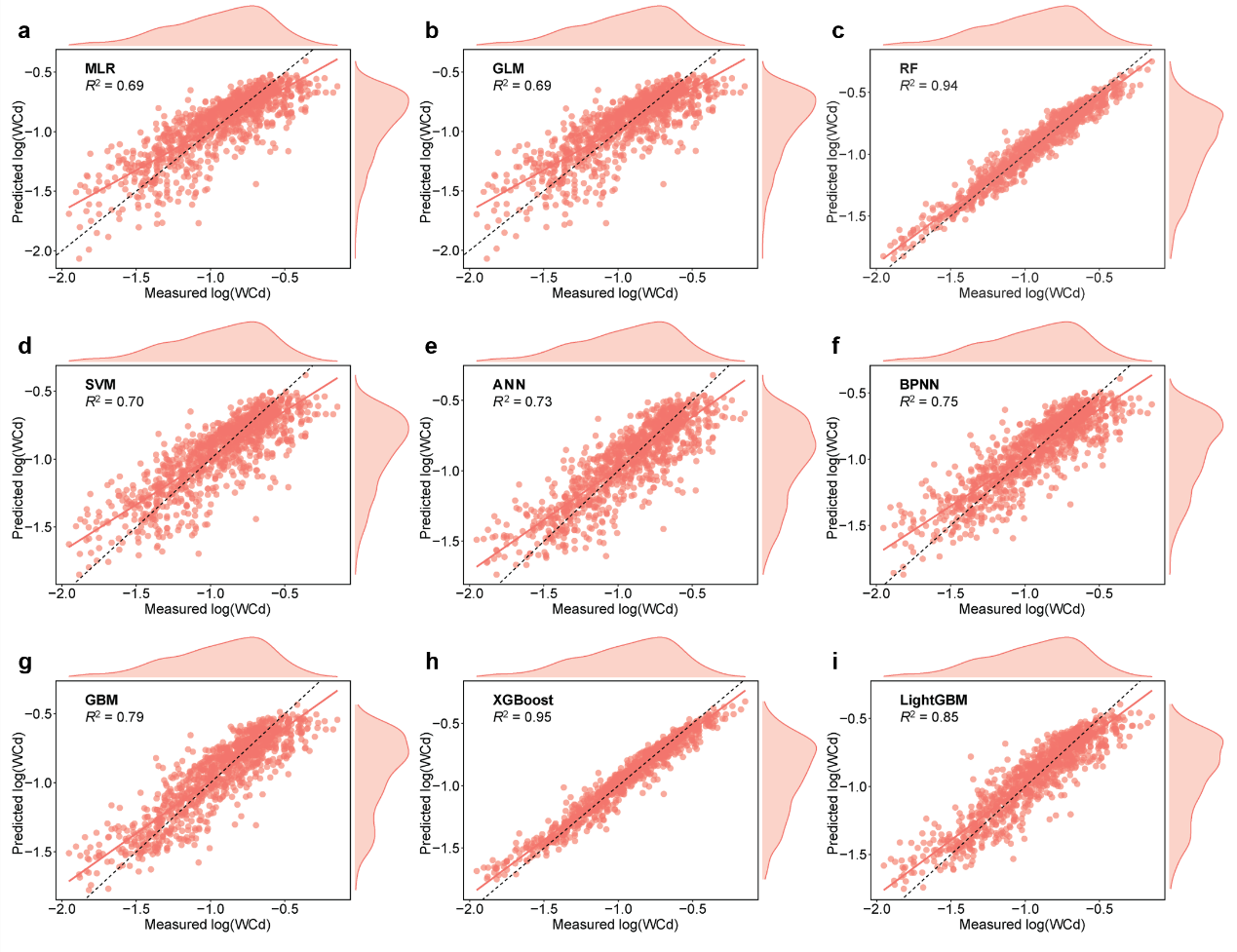


**Fig. S4.** Scatter plots of measured concentration versus predicted concentration in the log-transformed training datasets based on the nine models (n = 1,071).


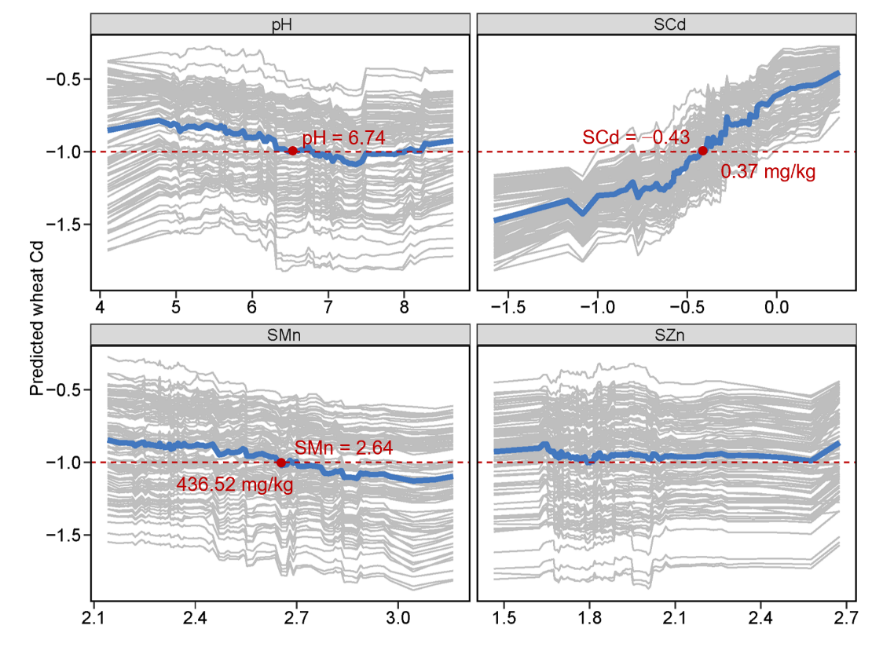


**Fig. S5.** The individual conditional expectation (ICE) plots in the XGBoost model. The red point is the critical value of each variable when the predicted wheat Cd concentration is -1.0 (i.e., 0.1 mg/kg).


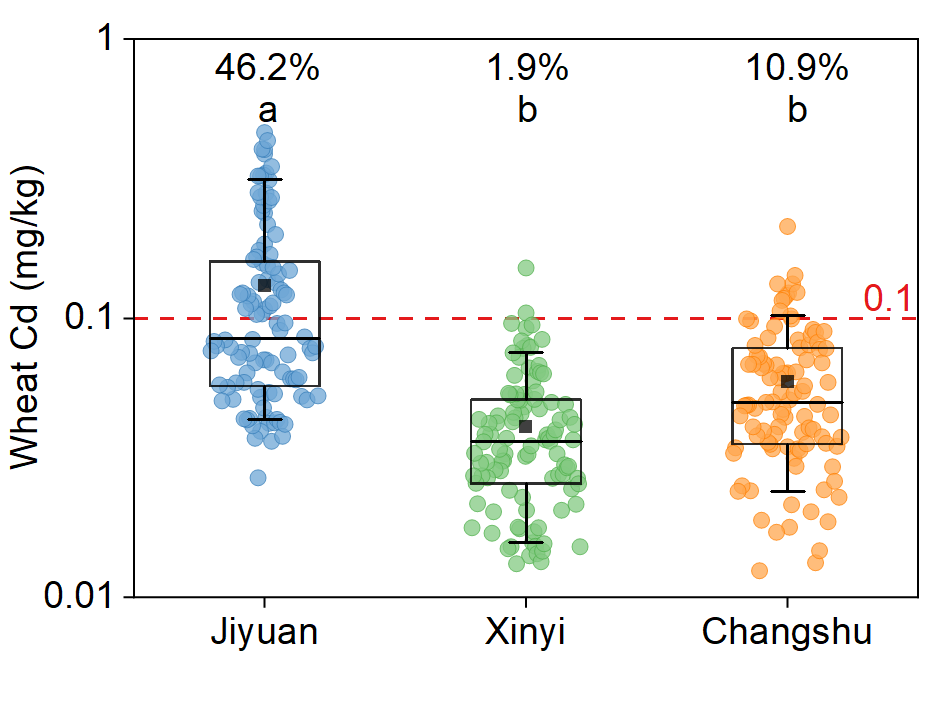


**Fig. S6.** Statistics plots of wheat grain Cd in regional surveys (n = 308). Squares represent mean values, solid lines indicate median values, and dotted lines denote standard limit values.


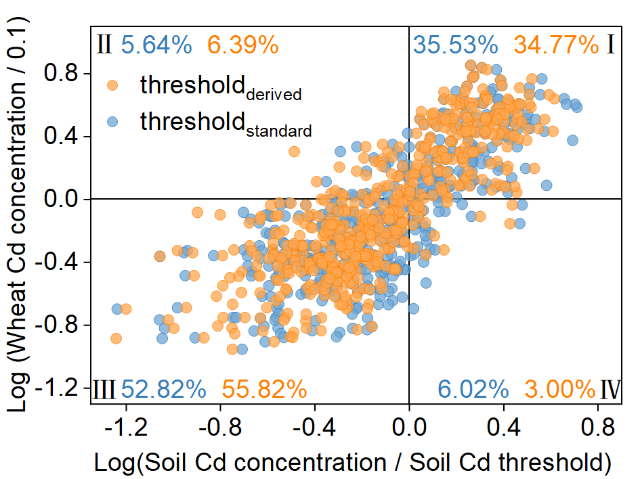


**Fig. S7.** The validity assessment of the soil thresholds (orange) versus risk screening values (blue, GB 15618-2018).


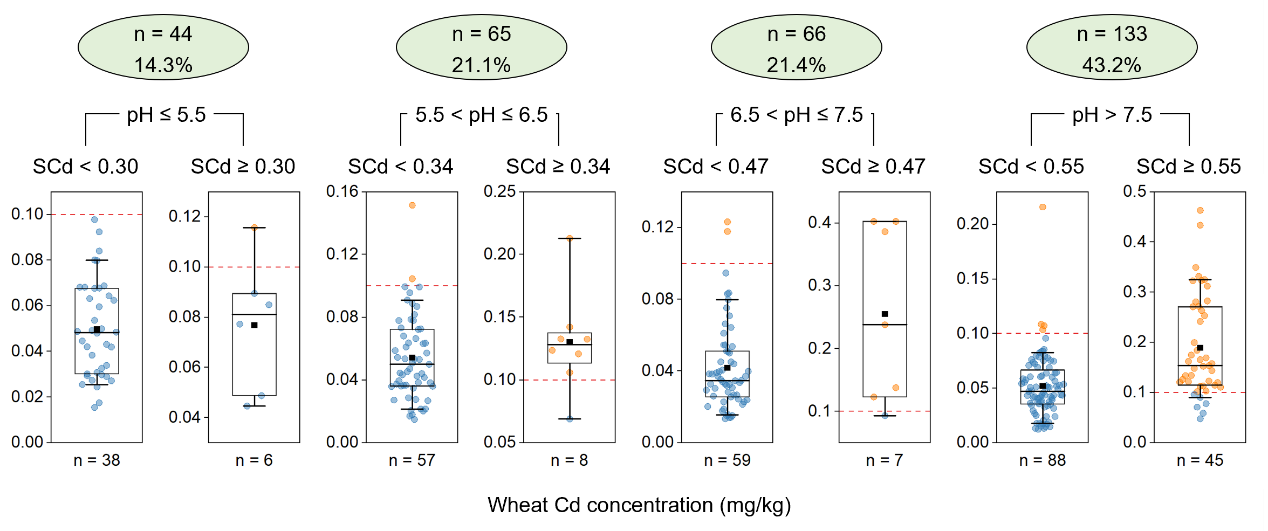


**Fig. S8.** Boxplot of wheat Cd concentration under different soil pH and soil Cd conditions (n = 308).

## Supplementary Tables:

**Table S1.** Treatment modes of field experiments in Changshu and Jiyuan.

|  | **Name** | **Optimized parameters** |
| --- | --- | --- |
| 1 | CK | control treatment |
| 2 | L | Lime CaCO_3_ (Target pH 6.5) |
| 3 | EF | Trace element fertilizer (Target pH 6.5) |
| 4 | Mn1 | MnSO_4_ fertilizer (5 kg Mn/Mu) |
| 5 | Mn2 | MnSO_4_ fertilizer (10 kg Mn/Mu) |
| 6 | Zn1 | ZnSO_4_ fertilizer (5 kg Zn/Mu) |
| 7 | Zn2 | ZnSO_4_ fertilizer (10 kg Zn/Mu) |
| 8 | OM | Organic matter (200 kg/Mu) |
| 9 | FZ | Foliar ZnSO_4_ (0.5% ZnSO_4_, 0.05% tween-20 v/v) |
| 10 | S | Na_2_SO_4_ (Sulfate equal to Zn1) |
| 11 | L-Zn1 | Lime CaCO_3_ (Target pH 6.5) + ZnSO_4_ fertilizer (5 kg Zn/Mu) |
| 12 | L-Mn1 | Lime CaCO_3_ (Target pH 6.5) + MnSO_4_ fertilizer (5 kg Mn/Mu) |
| 13 | L-FZ | Lime CaCO_3_ (Target pH 6.5) + Foliar ZnSO_4_ (0.5% ZnSO_4_, 0.05% tween-20 v/v) |

Note: The ‘Mu’ is a unit of area, about equal to 1/15 of a hectare or 666.67 m^2^.

**Table S2.** Characteristics of sampling sites for national-scale sampling survey in 2024.

| **Area** | **Province** | **Number** | **Soil Type** | **Climate Conditions** |
| --- | --- | --- | --- | --- |
| North China Huang-Huai region  (n = 84) | Hebei | 27 | Cinnamon Soils  Aquic soil | Temperate monsoon climate  12 ~ 15℃  500 ~ 800 mm |
|  | Shandong | 25 |  |  |
|  | Henan | 32 |  |  |
| Middle and Lower Reaches of the Yangtze River region  (n = 98) | Anhui | 27 | Paddy Soils  Red soil  Aquic soil  Yellow-Brown Soils | Subtropical monsoon climate  15 ~ 18℃  1000 ~ 1400 mm |
|  | Jiangsu | 40 |  |  |
|  | Zhejiang | 7 |  |  |
|  | Hubei | 24 |  |  |
| Southwest region  (n = 15) | Sichuan | 10 | Purple Soils  Yellow Soils | Humid subtropical climate  12 ~ 17℃  800 ~ 1100 mm |
|  | Yunnan | 2 |  |  |
|  | Guizhou &  Chongqing | 3 |  |  |

Note: Reference to Chinese Wheat Cultivation.

**Table S3.** Descriptive statistics of wheat Cd, soil metals, and soil physico-chemical properties (n = 1,339).

|  | **Unit** | **Mean** | **Median** | **SD** | **Min** | **Max** | **CV (%)** |
| --- | --- | --- | --- | --- | --- | --- | --- |
| Wheat Cd (WCd) | mg/kg | 0.15 | 0.13 | 0.10 | 0.01 | 0.71 | 68.9 |
| Soil Cd (SCd) | mg/kg | 0.55 | 0.53 | 0.40 | 0.03 | 2.23 | 72.9 |
| Soil Mn (SMn) | mg/kg | 391 | 377 | 250 | 138 | 1452 | 64.0 |
| Soil Cu (SCu) | mg/kg | 25.1 | 22.8 | 7.28 | 10.2 | 58.0 | 29.0 |
| Soil Zn (SZn) | mg/kg | 88.6 | 78.5 | 60.4 | 29.2 | 473. | 68.1 |
| Soil pH | - | 6.30 | 6.21 | 0.94 | 4.10 | 8.64 | 15.0 |
| Soil SOC | g/kg | 14.7 | 14.6 | 3.93 | 2.13 | 56.3 | 26.7 |
| Soil CEC ^a^ | cmol/kg | 16.5 | 16.3 | 0.88 | 14.2 | 23.3 | 5.40 |
| Soil sand ratio ^a^ | % | 15.5 | 13.5 | 3.70 | 8.60 | 32.8 | 23.8 |
| Soil clay ratio ^a^ | % | 23.4 | 25.3 | 3.60 | 15.3 | 30.7 | 15.2 |
| Soil silt ratio ^a^ | % | 61.1 | 61.2 | 2.50 | 44.4 | 67.9 | 4.20 |

^a^ Data from the Basic soil property dataset of high-resolution China Soil Information Grids.

**Table S4.** Descriptive statistics of train and test datasets for machine learning (n = 1,339).

|  | **Variables** | **Unit** | **Mean** | **Median** | **SD** | **Min** | **Max** | **CV (%)** |
| --- | --- | --- | --- | --- | --- | --- | --- | --- |
| Train  datasets  (n = 1,071) | Wheat grain Cd (WCd) | mg/kg | 0.15 | 0.13 | 0.10 | 0.01 | 0.71 | 67.6 |
|  | Soil total Cd (SCd) | mg/kg | 0.55 | 0.43 | 0.40 | 0.03 | 2.23 | 72.8 |
|  | Soil total Mn (SMn) | mg/kg | 388 | 372 | 249 | 138 | 1452 | 64.4 |
|  | Soil total Zn (SZn) | mg/kg | 88.3 | 78.2 | 59.5 | 29.2 | 458.8 | 67.4 |
|  | Soil pH | - | 6.29 | 6.21 | 0.94 | 4.10 | 8.64 | 15.0 |
| Test  datasets  (n = 268) | Wheat grain Cd (WCd) | mg/kg | 0.15 | 0.12 | 0.11 | 0.01 | 0.69 | 74.1 |
|  | Soil total Cd (SCd) | mg/kg | 0.53 | 0.43 | 0.39 | 0.03 | 1.93 | 73.4 |
|  | Soil total Mn (SMn) | mg/kg | 384 | 344 | 239 | 140 | 1398 | 62.2 |
|  | Soil total Zn (SZn) | mg/kg | 90.1 | 78.0 | 63.8 | 30.3 | 473.0 | 70.8 |
|  | Soil pH | - | 6.32 | 6.22 | 0.95 | 4.38 | 8.44 | 15.1 |

**Table S5.** The functions and optimized parameters of machine learning models.

|  | **Method** | **Function** | **Optimized parameters** |
| --- | --- | --- | --- |
| 1 | Generalized Linear Model  (GLM) | glm | family = gaussian |
| 2 | Random Forest  (RF) | randomForest | ntree = 1000  mtry = 3  nodesize = 5  nperm = 1 |
| 3 | Support Vector Machines  (SVM) | svm | cost = 1  gamma = 0.01  method = radial |
| 4 | Artificial Neural Network  (ANN) | nnet | size = 5  decay = 0.01  maxit = 1000  rang = 0.1 |
| 5 | Back Propagation Neural Network  (BPNN) | neuralnet | hidden = c (5, 3)  threshold = 0.01  learningrate = 0.01  linear.output = T |
| 6 | Gradient Boosting Machine  (GBM) | gbm | n.trees = 1000  interaction.depth = 3  shrinkage = 0.01  distribution = gaussian |
| 7 | Extreme Gradient Boosting  (XGBoost) | xgboost | eta = 0.01  nrounds = 1000  subsample = 0.8  booster = gbtree  colsample_bytree=0.8  objective = reg:squarederror |
| 8 | Light Gradient Boosting Machine  (LightGBM) | lightgbm | num_leaves = 10  earning_rate = 0.01  nrounds = 1000  objective = regression |

**Table S6.** Performance of the 5-fold cross validation during model training.

|  | **Method** | Fold 1  (R^2^) | Fold 2  (R^2^) | Fold 3  (R^2^) | Fold 4  (R^2^) | Fold 5  (R^2^) |
| --- | --- | --- | --- | --- | --- | --- |
| 1 | Multiple Linear Regression  (MLR) | 0.65 | 0.69 | 0.70 | 0.69 | 0.72 |
| 2 | Generalized Linear Model  (GLM) | 0.66 | 0.69 | 0.71 | 0.67 | 0.72 |
| 3 | Random Forest  (RF) | 0.92 | 0.95 | 0.94 | 0.93 | 0.94 |
| 4 | Support Vector Machines  (SVM) | 0.70 | 0.73 | 0.70 | 0.67 | 0.68 |
| 5 | Artificial Neural Network  (ANN) | 0.73 | 0.71 | 0.72 | 0.73 | 0.75 |
| 6 | Back Propagation Neural Network  (BPNN) | 0.71 | 0.75 | 0.75 | 0.77 | 0.76 |
| 7 | Gradient Boosting Machine  (GBM) | 0.82 | 0.78 | 0.79 | 0.81 | 0.75 |
| 8 | Extreme Gradient Boosting  (XGBoost) | 0.95 | 0.95 | 0.93 | 0.96 | 0.96 |
| 9 | Light Gradient Boosting Machine  (LightGBM) | 0.88 | 0.85 | 0.84 | 0.82 | 0.85 |

**Table S7.** Descriptive statistics of wheat-producing region in China (n = 373).

|  | **Variables** | **Unit** | **Mean** | **Median** | **SD** | **Min** | **Max** | **CV (%)** |
| --- | --- | --- | --- | --- | --- | --- | --- | --- |
| Sampling  (n = 197) | Wheat grain Cd (WCd) | mg/kg | 0.05 | 0.04 | 0.03 | 0.01 | 0.17 | 67.3 |
|  | Soil total Cd (SCd) | mg/kg | 0.19 | 0.17 | 0.13 | 0.05 | 0.66 | 65.3 |
|  | Soil total Mn (SMn) | mg/kg | 473 | 462 | 151 | 152 | 859 | 30.8 |
|  | Soil total Zn (SZn) | mg/kg | 82.7 | 76.9 | 26.3 | 41.0 | 172.5 | 31.8 |
|  | Soil pH | - | 6.88 | 7.05 | 0.96 | 4.48 | 8.82 | 13.9 |
| Published  research  (n = 176) | Wheat grain Cd (WCd) | mg/kg | 0.10 | 0.09 | 0.09 | 0.01 | 0.58 | 92.5 |
|  | Soil total Cd (SCd) | mg/kg | 0.65 | 0.32 | 0.79 | 0.02 | 3.85 | 120.8 |
|  | Soil total Mn (SMn) | mg/kg | 489 | 424 | 215 | 217 | 676 | 44.1 |
|  | Soil total Zn (SZn) | mg/kg | 92.7 | 90.0 | 31.0 | 23.3 | 288.4 | 33.5 |
|  | Soil pH | - | 7.39 | 7.67 | 0.95 | 4.67 | 8.90 | 12.9 |

**Table S8.** Linear regression models to predict wheat grain Cd concentrations (n = 308).

|  | **Model** | **R^2^** | **F** | **P** |
| --- | --- | --- | --- | --- |
| Eq.1 | Log(WCd) = 0.73Log(SCd) – 0.67 | 0.48 | 384.6 | <0.01 |
| Eq.2 | Log(WCd) = 0.78Log(SCd) – 0.14pH + 0.25 | 0.65 | 202.6 | <0.01 |
| Eq.3 | Log(WCd) = 0.77Log(SCd) – 0.11pH – 0.30Log(SMn) + 0.78 | 0.69 | 149.8 | <0.01 |
| Eq.4 | Log(WCd) = 0.77Log(SCd) – 0.11pH – 0.30Log(SMn) – 0.01 Log(SZn) + 0.78 | 0.70 | 147.1 | <0.01 |

**Table S9.** Comparison of model performance based on test datasets (n = 268).

|  | **Model** | **Slope** | **R^2^** | **RMSE** | **Bias** |
| --- | --- | --- | --- | --- | --- |
| 1 | Multiple Linear Regression (MLR) | 0.55 | 0.55 | 0.183 | 0.022 |
| 2 | Generalized Linear Model (GLM) | 0.54 | 0.56 | 0.180 | 0.021 |
| 3 | Random Forest (RF) | 0.74 | 0.85 | 0.171 | 0.013 |
| 4 | Support Vector Machines (SVM) | 0.52 | 0.59 | 0.178 | 0.019 |
| 5 | Artificial Neural Network (ANN) | 0.60 | 0.60 | 0.173 | 0.016 |
| 6 | Back Propagation Neural Network (BPNN) | 0.57 | 0.61 | 0.175 | 0.017 |
| 7 | Gradient Boosting Machine (GBM) | 0.60 | 0.67 | 0.171 | 0.014 |
| 8 | Extreme Gradient Boosting (XGBoost) | 0.76 | 0.88 | 0.169 | 0.012 |
| 9 | Light Gradient Boosting Machine (LightGBM) | 0.67 | 0.77 | 0.173 | 0.014 |

Note: RMSE is the root mean square error.

**Table S10.** Soil Cd threshold for wheat safe production.

| pH | Soil Cd threshold (mg/kg) | |
| --- | --- | --- |
|  | Derived value  by XGBoost model | Risk screening value  (GB15618-2018) |
| pH ≤ 5.5 | 0.30 (0.28 - 0.32) | 0.30 |
| 5.5 < pH ≤ 6.5 | 0.34 (0.32 - 0.37) | 0.30 |
| 6.5 < pH ≤ 7.5 | 0.47 (0.38 - 0.53) | 0.30 |
| pH > 7.5 | 0.55 (0.51 - 0.59) | 0.60 |

Note: Values in parentheses are 95% confidence intervals for soil Cd threshold.
